# Intubation conditions and neonatal outcomes with rocuronium versus suxamethonium in cesarean sections: A systematic review and meta-analysis

**DOI:** 10.1186/s12871-025-03321-6

**Published:** 2025-10-02

**Authors:** Atef A. Hassan, Mohamed A. Khalafallah, Noha Rami Ismail, Esraa Menshawy, Abdulrhman Mady, Moaz Abouelmagd, Amr Menshawy, Ahmed Mensahwy

**Affiliations:** 1 Faculty of Medicine, Al-Azhar University, Cairo, Egypt; 2 Faculty of Medicine, Alexandria University, Alexandria, Egypt; 3 Faculty of Medicine, Zagazig University, Zagazig, Egypt; 4 Faculty of Medicine, Department of Obstetrics and Gynecology, Al Zahraa Hospital, Al-Azhar University, Cairo, Egypt; 5 Faculty of Medicine, Elrazi University, Khartoum, Sudan; 6 Faculty of Medicine, Cairo University, Cairo, Egypt; 7 Department of Obstetrics and Gynecology, Faculty of Medicine, Al- Azhar University, Cairo, Egypt

**Keywords:** Rocuronium, Succinylcholine, Apgar score, Cesarean section, Neonatal outcomes

## Abstract

**Background:**

While general anesthesia is necessary for some emergency deliveries, it carries risks such as failed intubation and neonatal complications. This study investigates whether rocuronium and suxamethonium (also known as Succinylcholine) provide equivalent conditions for airway management and neonatal outcomes during cesarean sections. Ensuring the safety and efficacy of these agents is crucial due to the physiological and hemodynamic changes associated with pregnancy and delivery.

**Method:**

We conducted a systematic review and meta-analysis following PRISMA guidelines. We included studies involving pregnant women undergoing cesarean sections that used rocuronium as an intervention and suxamethonium as a comparator, reporting outcomes such as Apgar scores, surgery duration, and time-related metrics. A comprehensive search of databases was performed up to July 2025. Statistical analyses were performed using RevMan Software, assessing heterogeneity and summarizing findings through mean differences and risk ratios.

**Results:**

Our metaanalysis of six studies comprising 1,122 patients showed that succinylcholine significantly reduced the time from induction to umbilical cord clamping compared with rocuronium (mean difference [MD] 19.21 s; 95% CI 5.15 to 33.27; *P* = 0.007; I² = 0%). There was no significant difference between groups in time from incision to delivery (MD − 12.72 s; 95% CI − 84.84 to 59.41; *P* = 0.73; I² = 94%) or in total surgery duration (MD − 1.20 min; 95% CI − 3.70 to 1.30; *P* = 0.34; I² = 63%). Newborns in the succinylcholine group were more likely to achieve favorable 1 min Apgar scores (risk ratio [RR] 1.87; 95% CI 1.31 to 2.67; *P* = 0.0006; I² = 0%) and 5 min Apgar scores (RR 2.52; 95% CI 1.21 to 5.25; *P* = 0.01; I² = 30%), whereas 10 min Apgar scores did not differ significantly (RR 2.51; 95% CI 0.66 to 9.64; *P* = 0.18; I² = 34%). In the subgroup of scheduled cesarean deliveries, only the 1 min Apgar score remained significantly higher with succinylcholine (RR 1.75; 95% CI 1.10 to 2.77; *P* = 0.02; I² = 0%).

**Conclusion:**

This review suggests that suxamethonium offers benefits like shorter induction-to-cord clamping times and better early Apgar scores. The current evidence is still limited and further well-designed randomized controlled trials are needed to explore the relationship between rocuronium levels and neonatal outcomes.

**Supplementary Information:**

The online version contains supplementary material available at 10.1186/s12871-025-03321-6.

## Introduction

Globally, it has been reported that 21.1% of women deliver by cesarean section (CS) [1]. The American Society of Anesthesiologists recommends neuraxial anesthesia as the gold standard for CS because it is generally regarded as safer than general anesthesia [2]. General anesthesia poses substantial risks, including failed intubation, aspiration of gastric contents, increased blood loss, awareness, and neonatal respiratory complication [3, 4]. Variable indications for general anesthesia for patients undergoing CS have been reported including, fetal or maternal clinical emergency, maternal anxiety, neuraxial block failure, complications or contraindications [4].

Although there is a substantial amount of non-obstetric literature on airway management during general anesthesia, some of which can be extrapolated to the obstetric situation, our literature search showed that few obstetric studies exist. A systematic review and meta-analysis analyzed the results of 50 trials in 4151 patients undergoing general anesthesia, showing that suxamethonium (also known as Succinylcholine) (SUX) provides better intubation circumstances over rocuronium (ROC) at doses of 0.6–0.7 mg/kg but no statistically significant superiority at higher doses of ROC 0.9–1.2 mg/kg [5].

Despite decades of clinical experience, no prior systematic review has synthesized randomized and observational evidence on how SUX and ROC compare for both airway conditions and neonatal outcomes in cesarean delivery. Given that even small differences in induction‑to‑delivery intervals or Apgar scores can influence neonatal acid–base status and long‑term neurodevelopment, providing clear, evidence‑based guidance is essential for both elective and emergency CS [6].

This meta‑analysis aims to determine whether succinylcholine and rocuronium yield comparable intubation conditions and neonatal outcomes in women undergoing cesarean delivery under general anesthesia. By quantifying differences in induction‑to‑delivery times, Apgar scores, and maternal intubation conditions, we seek to inform clinical protocols and support anesthesiologists in selecting the agent that optimally balances speed, safety, and neonatal health.

## Methods

Following PRISMA guidelines, we conducted a systematic review and meta-analysis [7]. We have registered the protocol in PROSPERO under the registration number CRD420251086127.

### Eligibility criteria

The following conditions were considered for the study:

1. Studies whose population includes pregnant women undergoing CS.
2. Studies that used ROC as an intervention are eligible.
3. Studies that used SUX as a comparator are eligible.
4. Studies reporting any one of the following outcomes: intubation conditions, 1, 5, 10 min Apgar scores, surgery duration, time from induction to clamping the umbilical cord, time from incision to delivery, and time from surgical skin closure to tracheal extubation are eligible.
5. Studies described as randomized clinical trials (RCTs) or Quasi-Experimental.

We excluded studies that were not available in English, conference abstracts, and single-arm. In addition, studies that did not include pregnant women undergoing CS.

### Literature search strategy

We performed a computer literature search of Web of Science, PubMed, Cochrane CENTRAL, and Scopus from inception until July 2025 using the following query: (“Rocuronium“[Mesh] OR rocuronium OR “Rocuronium Bromide”) AND (“Succinylcholine“[Mesh] OR suxamethonium OR “Suxamethonium Chloride”) AND (“Cesarean Section“[Mesh] OR “Cesarean Delivery” OR “C-section” OR “Caesarean”). The specific search strategy used for each database is detailed in Supplementary File 1.

### Selection of studies

Two authors independently screened titles and abstracts and then retrieved the full text of selected studies to evaluate their applicability and validity. Disagreements were resolved through a third author.

### Data extraction

Three independent reviewers systematically extracted data using a standardized Microsoft Excel sheet. For each included trial, we recorded key study design elements, population characteristics, and risk‑of‑bias assessments, and we captured all outcomes reported in sufficient detail for meta‑analysis. Maternal and neonatal endpoints, including the time from induction to umbilical‑cord clamping, the time from skin incision to delivery, total surgery duration, newborn birth weight, and gestational age; neonatal Apgar scores at 1, 5, and 10 min; maternal intubation‑condition scores classified as excellent, good, or poor; and umbilical arterial blood‑gas parameters; pH, PCO₂, PO₂, and base excess (BE).

### Assessment risk of bias

The risk of bias was evaluated by two independent reviewers using the Cochrane Risk of Bias assessment tool (ROB 2.0) for RCTs. Each study was assessed for potential bias in these domains: (1) random sequence generation, (2) allocation concealment, (3) blinding of participants, personnel, and outcome assessors, (4) incomplete outcome data, (5) selective outcome data reporting, and (6) other sources of bias. Any disagreements were resolved by a third author.

### Data synthesis

For continuous data, we used the mean difference (MD) with its corresponding 95% confidence interval. Dichotomous outcomes were analyzed using the risk ratio (RR) and its 95% confidence interval. We assessed heterogeneity among the included studies using the chi-square test and I2.

to detect the amount of it. All statistical analyses were conducted using RevMan Software.

## Results

### Characteristics of the included studies

**Fig. 1:**
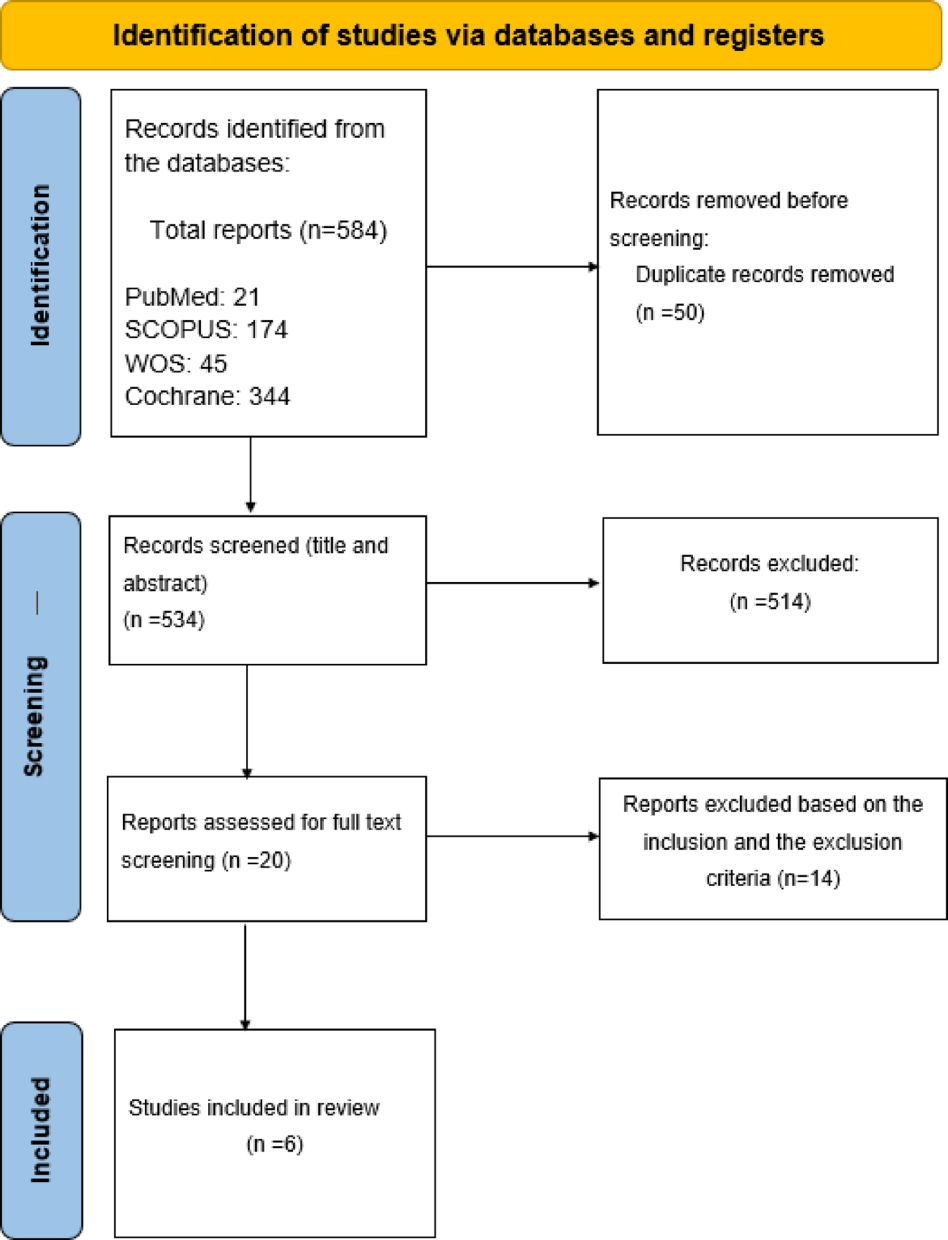
PRISMA flow diagram of the studies selection process

Six studies were included in our review of the initially screened 584 records (Fig. 1), compromising evidence from 1122 patients [8–13]. Five of them were RCTs and one study had a quasi-experimental design comparing ROC (1 mg/kg) with SUX (1 mg/kg) for general anesthesia in CS (Table 1). Three of the included studies had a low risk of bias. In contrast, the rest had some concerns about their risk of bias indicating caution in interpreting their results (Table 2). In the included studies, the mean age for participants ranged between 31 and 34 years while their BMI ranged from 28.0 to 30.0 kg/m². Additionally, most patients fell into the ASA PS Score I or II categories. For further details, refer to Table 3.

**Table 1:** Summary of the studies comparing Rocuronium and suxamethonium in Cesarean section

Study ID	Title	Design	Location	Population	Intervention	Comparator	Follow Up	Sample Size	Aim	Conclusion
Stourac 2016	Low-Dose or High-Dose Rocuronium Reversed with Neostigmine or Sugammadex for Cesarean Delivery Anesthesia	Randomized single-blinded (parturient) parallel-group controlled study	University hospitals (Brno, Olomouc, Czech Republic)	Women aged 14–60 years admitted for cesarean delivery	ROC: Rocuronium 1 mg/kg	SUX: Succinylcholine 1 mg/kg	24 h post-operation	240	To determine if sugammadex can reverse deep neuromuscular blockade for intubation in cesarean delivery patients	Rocuronium is more effective for intubation, less likely to cause laryngoscopy resistance and myalgia compared to suxamethonium
Bla´ha 2020	Surgical conditions with rocuronium versus suxamethonium in cesarean section	Prospective randomized double-blind controlled trial	A tertiary care university hospital	Women aged 18–45 with good physical status, written informed consent, difficult fetal delivery for cesarean sections under general anesthesia	ROC: Rocuronium 0.6 mg/kg; sugammadex 4 mg/kg for reversal	SUX: Suxamethonium 1 mg/kg; atracurium 0.25 mg/kg post-delivery; neostigmine with atropine for reversal	24 h post-surgery	90	To compare surgical conditions for fetal delivery during cesarean section under general anesthesia between rocuronium and suxamethonium	Rocuronium allows better surgical conditions, easier delivery, and shorter incision-to-delivery interval
Ahad 2018	Comparison of succinylcholine and rocuronium for rapid sequence intubation in cesarean section	Randomized controlled trial	Department of Anesthesia, SICU & Pain Center, People’s Medical College Hospital, Nawabshah	Full-term parturient aged 30–35 years, Mallampati class I & II, ASA class I and II	ROC: Rocuronium 1 mg/kg	SUX: Succinylcholine 1 mg/kg	N/A	124	To compare intubating conditions between rocuronium and succinylcholine for rapid sequence induction in cesarean section	Rocuronium provides similar intubating conditions to succinylcholine and can be safely used for rapid sequence induction
Kosinova 2017	Rocuronium versus suxamethonium for rapid sequence induction of general anaesthesia for caesarean section	Randomized single-blinded parallel-group controlled study	University Hospital Brno and University Hospital Olomouc	488 parturients aged 14–60 years with 525 newborns; exclusions included allergies, intolerance to drugs, iodine reactions, patient refusal, or missing Apgar score data	ROC: Rocuronium 1 mg/kg	SUX: Suxamethonium 1 mg/kg	N/A	488 parturients (525 newborns)	To explore the difference in Apgar scores between rocuronium and suxamethonium for rapid sequence induction	Rocuronium linked to lower Apgar scores at 1 min compared to suxamethonium, clinical significance unclear
Abu-Halweh 2007	Rapid sequence induction and intubation with 1 mg/kg rocuronium bromide in cesarean section	Randomized controlled trial	Jordan University Hospital	20 patients, ASA I-II, undergoing elective or emergency cesarean section	ROC: Thiopentone 5 mg/kg + Rocuronium 1 mg/kg	SUX: Thiopentone 5 mg/kg + Suxamethonium 1 mg/kg	N/A	120	To assess intubating conditions with Rocuronium Bromide 1 mg/kg and Thiopentone 5 mg/kg in elective or emergency cesarean sections	Rocuronium Bromide 1 mg/kg provides similar intubating conditions to suxamethonium with no significant differences
SHEIKH N A 2008	Comparison of Intubating Conditions Produced by Rocuronium and Suxamethonium for Rapid Sequence Induction in Elective Caesarean	Quasi-experimental study	Operative rooms of Hameed Latif Hospital, Lahore	50 full-term pregnant patients for elective caesarean section; exclusions included preterm labor, difficult intubation, neuromuscular disease, malignant hyperthermia, drug interactions, and Rocuronium allergy	ROC: Thiopentone 5 mg/kg + Rocuronium 1 mg/kg	SUX: Thiopentone 5 mg/kg + Suxamethonium 1.5 mg/kg	N/A	50	To determine if Rocuronium and Suxamethonium provide equally good intubating conditions in elective caesarean sections using RSI	Rocuronium 1 mg/kg provides equally good intubating conditions compared to suxamethonium 1.5 mg/kg in elective caesarean section using RSI at 60 s

ROC: Rocuronium, SUX: Suxamethonium, RSI: Rapid sequence induction, N/A: Not Available, ASA PS: American Society of Anesthesiologists Physical Status

**Table 2:** Risk of bias

Study ID	Randomization Process	Deviations from Intended Interventions	Missing Outcome Data	Measurement of the Outcome	Selection of the Reported Result	Overall Bias
Stourac 2016	Low	Low	Low	Low	Low	Low
Bla´ha 2020	Low	Some concerns	Low	Low	Low	Some concerns
Ahad 2018	Some concerns	Low	Low	Some concerns	Low	Some concerns
Kosinova 2017	Low	Low	Low	Low	Low	Low
Abu-Halweh 2007	Low	Low	Low	Low	Low	Low
SHEIKH N A 2008	Some concerns	Low	Low	Some concerns	Low	Some concerns

**Table 3:** Baseline characteristics of studies comparing Rocuronium and suxamethonium in Cesarean sections

Study ID	Groups	Age (mean ± SD)	BMI at Delivery (kg/m², mean ± SD)	Gestational Age (wk, mean ± SD)	ASA PS Score I (%)	ASA PS Score II (%)	ASA PS Score III (%)	ASA PS Score IV (%)
Stourac 2016	ROC	31 (5)	30 (6)	N/A	105(88%)	105(88%)	15(13%)	15(13%)
	SUX	31 (5)	30 (6)	N/A	113(94%)	113(94%)	7(6%)	7(6%)
Bla´ha 2020	ROC	32.5 (2.2)	28.0 (4.6)	168.3 (6.4)	N/A	N/A	N/A	N/A
	SUX	34.0 (5.5)	29.9 (4.8)	166.6 (7.9)	N/A	N/A	N/A	N/A
Ahad 2018	SUX	32.87 (1.94)	N/A	N/A	39 (62.9%)	23 (37.10%)	N/A	N/A
	ROC	31.87 (1.91)	N/A	N/A	34 (54.84%	28 (45.16%	N/A	N/A
Kosinova 2017	ROC	31 (5.3)	30 (5.6)	166 (6.4)	118 (42.2%)	105 (42.9%)	14 (5.7%)	8 (3.3%)
	SUX	31 (5.2)	30 (5.9)	165 (7.1)	116 (47.7%)	109 (44.9%)	12 (4.9%)	6 (2.5%)
Abu-Halweh 2007	ROC	33.1 (4.9)	N/A	N/A	50(83%)	10(17%)	N/A	N/A
	SUX	31.2 (6.5)	N/A	N/A	57(95%)	3(5%)	N/A	N/A

Note: ROC: Rocuronium, SUX: Suxamethonium, ASA PS: American Society of Anesthesiologists Physical Status, BMI: Body Mass Index, N/A: Not Available

### Quality assessment

The overall quality assessment is three RCTs with a low risk of bias and three studies with some concern risk or bias, two RCTs and one Quasi-Experimental. All details are presented in Table 2.

### Meta-analysis of time from induction to clamping the umbilical cord(s)

**Fig. 2:**
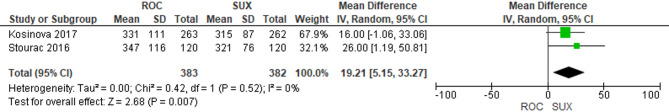
Meta-analysis of time from induction to clamping the umbilical cord (s)

Two studies were included in the meta-analysis comparing ROC (*n* = 383) and SUX (*n* = 382). The time from induction to clamping the umbilical cord is shorter in the SUX group compared to the ROC group (MD = 19.21; 95% CI= [5.15, 33.27]; *P* = 0.007; I²=0%) (Fig. 2).

### Time from incision to delivery and surgery duration

**Fig. 3:**
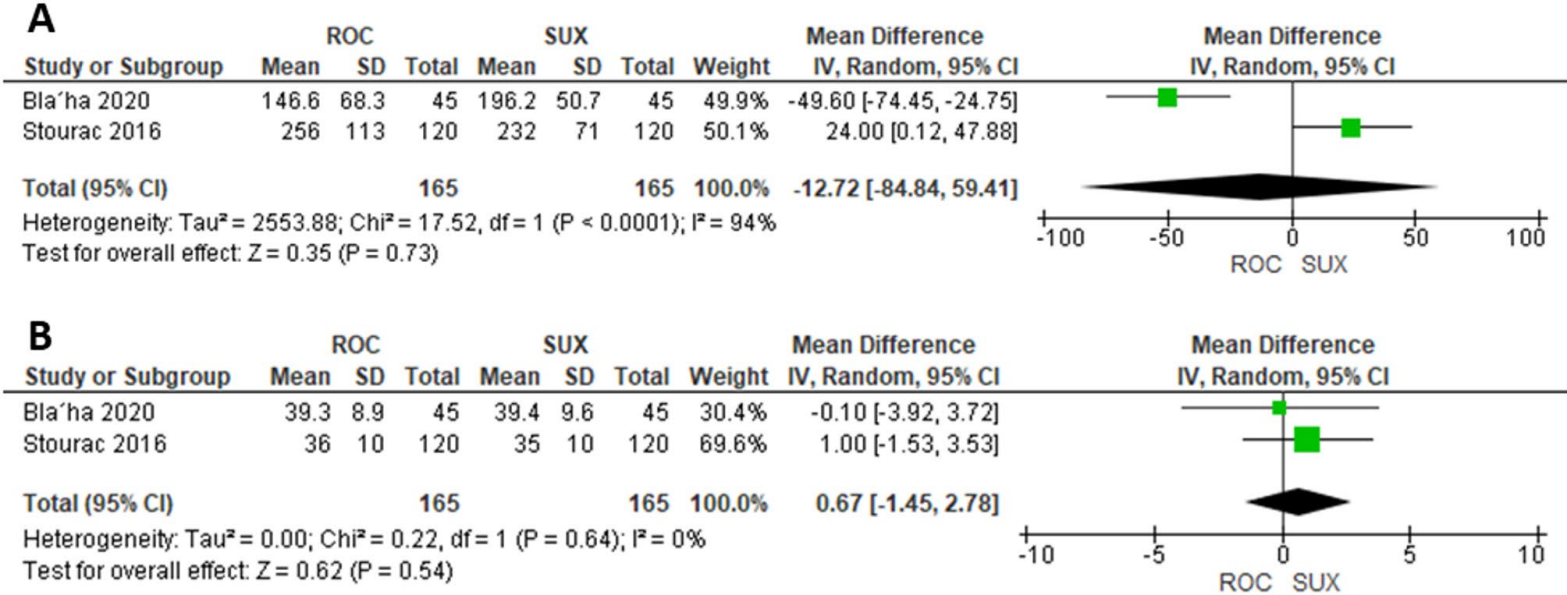
**A**) Time from incision to delivery, **B**) Surgery duration

Two studies were included in the meta-analysis comparing ROC (*n* = 165) and SUX (*n* = 165). The mean time from incision to delivery did not differ significantly between the ROC and SUX groups (MD= −12.72 s, 95% CI= [−84.84, 59.41]; *P* = 0.73; I²=94%) (Fig. 3A). Neither did the mean Surgery duration did not differ significantly between the ROC and SUX groups (MD= −1.2 min, 95% CI= [−3.7, 1.3]; *P* = 0.34; I²=63%) (Fig. 3B).

### Newborn weight and gestational age

**Fig. 4:**
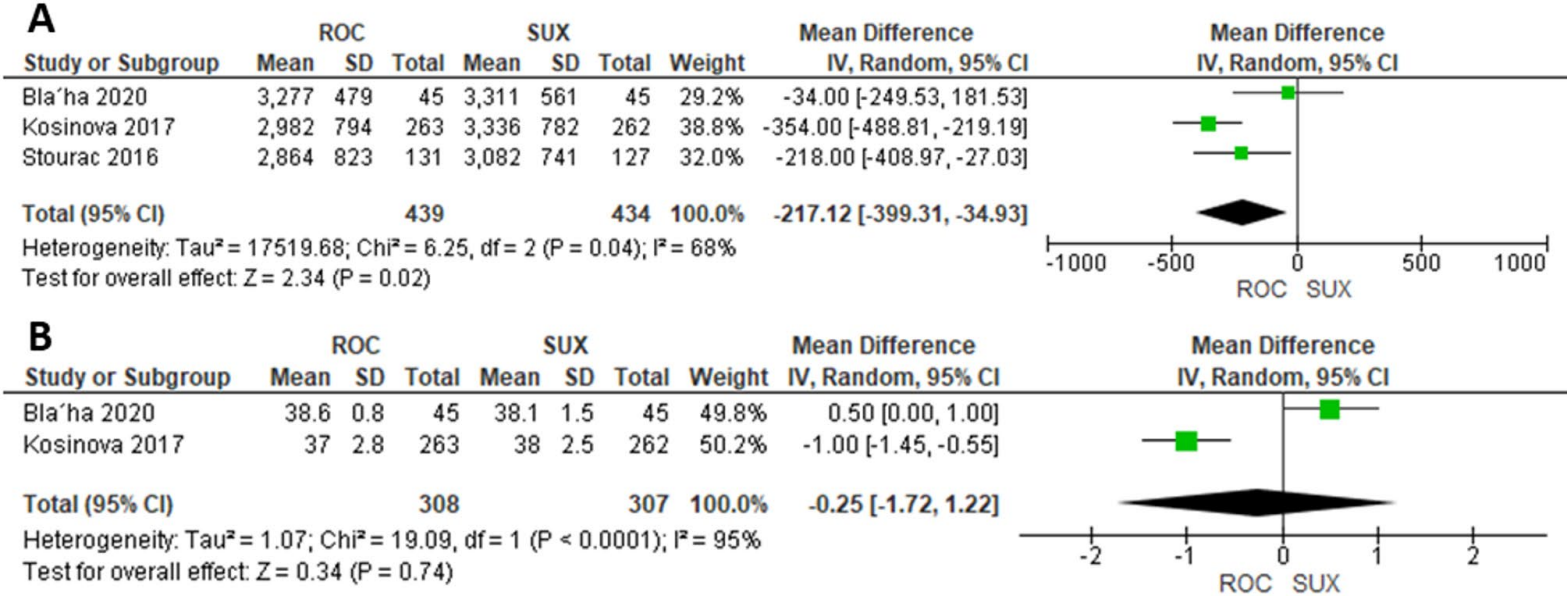
**A**) Newborn weight, **B**) Gestational age

Three studies were included in the meta-analysis comparing ROC (*n* = 439) and SUX (*n* = 434) for newborn weight that was significantly lower in the ROC group compared to the SUX group (MD= −217.12 g, 95% CI= [−399.31, −34.93]; *P* = 0.02; I²=68%) (Fig. 4A). Two studies compared ROC (*n* = 308) and SUX (*n* = 307) for gestational age and found that it did not differ significantly between the groups (MD= −0.25 weeks, 95% CI= [−1.72, 1.22]; *P* = 0.74; I²=95%) (Fig. 4B).

### Meta-analysis of APGAR scores 1, 5, and 10 min

**Fig. 5:**
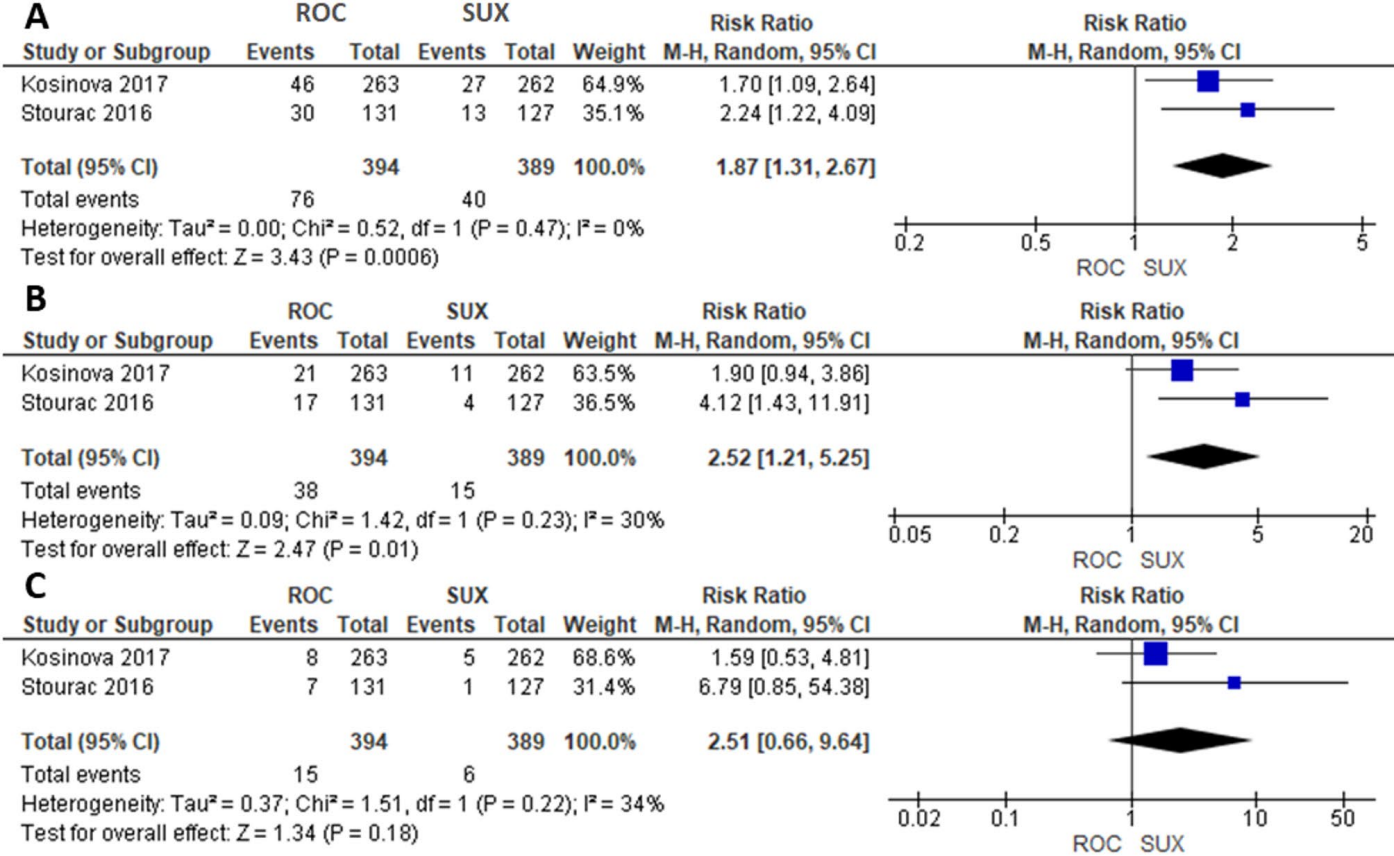
**A**) 1-minute Apgar score, **B**) 5-minute Apgar score, **C**) 10-minute Apgar score

**Fig. 6:**
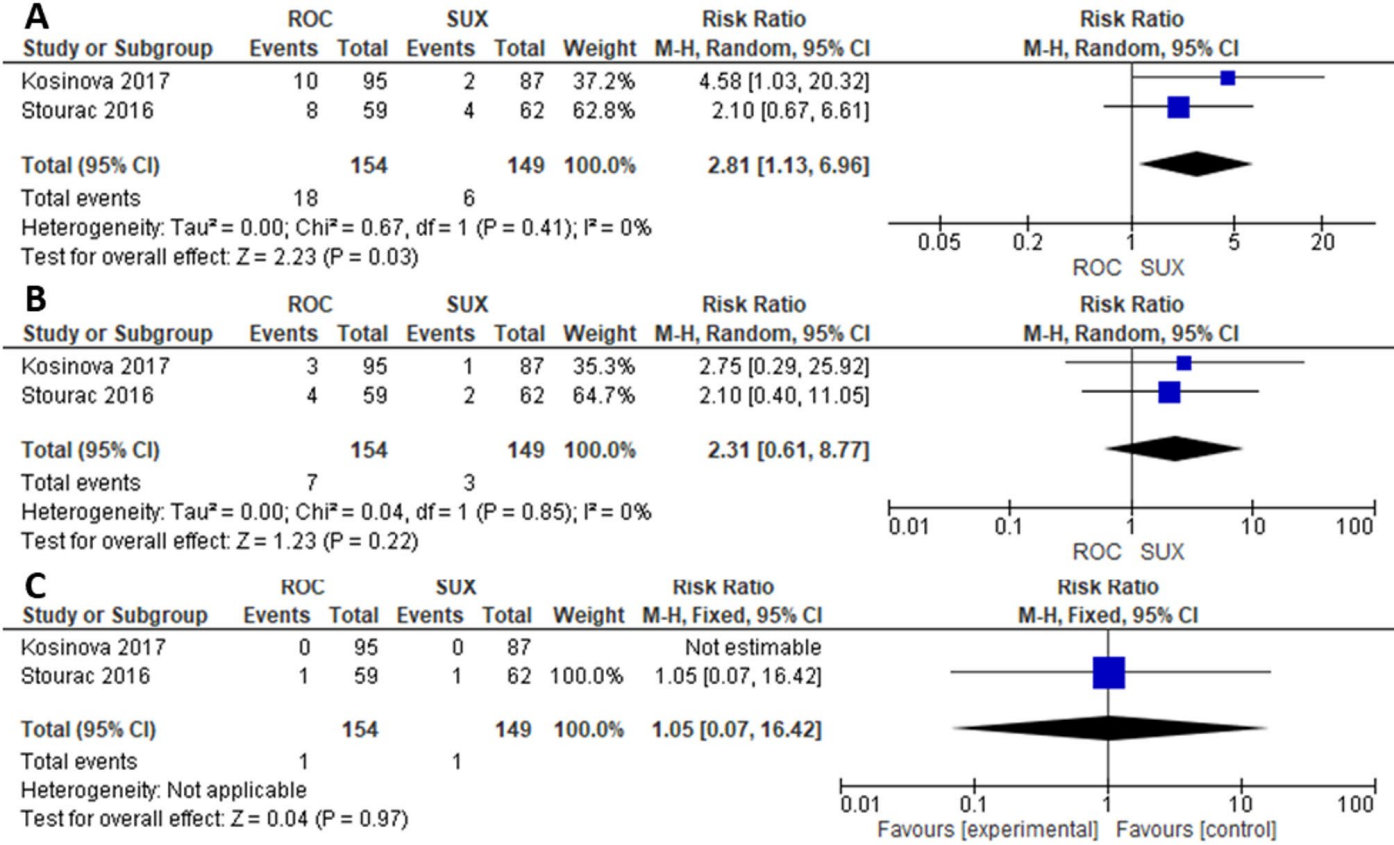
Scheduled CS **A**) 1-minute Apgar score, **B**) 5-minute Apgar score, **C**) 10-minute Apgar score

Two studies were included in the meta-analysis comparing ROC (*n* = 394) and SUX (*n* = 389). The risk of a favorable 1-min and 5-min Apgar score was significantly higher in the SUX group compared to the ROC group (RR = 1.87; 95% CI= [1.31, 2.67]; *P* = 0.0006; I²=0%) (Fig. 5A), (RR = 2.52; 95% CI= [1.21, 5.25]; *P* = 0.01; I²=30%) (Fig. 5B). While the risk of a favorable 10-min Apgar score did not differ significantly between the ROC and SUX groups (RR = 2.51; 95% CI= [0.66, 9.64]; *P* = 0.18; I²=34%) (Fig. 5C). However, in the case of scheduled CS deliveries, only a 1-min Apgar score was significant in favor of SUX (Fig. 6).

### Meta-analysis of intubation conditions

**Fig. 7:**
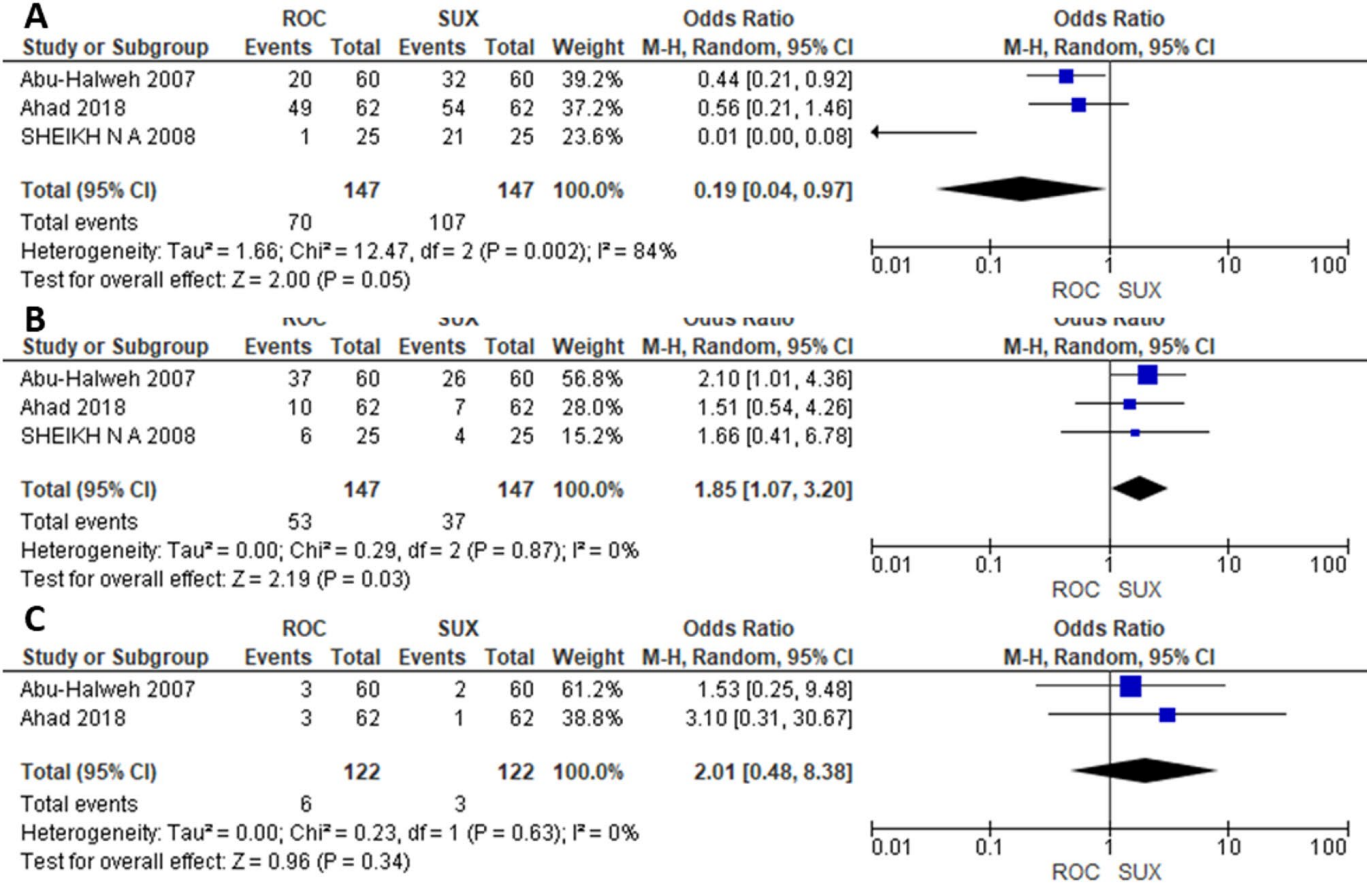
**A**) Excellent conditions, **B**) good conditions, **C**) poor conditions

Three studies were included in the meta-analysis comparing ROC (*n* = 147) and SUX (*n* = 147) for excellent and good intubation conditions. The odds of achieving excellent intubation conditions were significantly lower in the ROC group (OR = 0.19; 95% CI= [0.04, 0.97]; *P* = 0.05; I²=84%). In contrast, the odds of achieving good intubation conditions were lower in the SUX group (OR = 1.85; 95% CI= [1.07, 3.20]; *P* = 0.03; I²=0%), respectively (Fig. 7A, B). Additionally, only two studies contributed to the meta-analysis of poor intubation conditions, which revealed no significant difference between the Rocuronium and Succinylcholine groups (OR = 2.01; 95% CI = [0.48, 8.38]; *P* = 0.34; I² = 0%). (Fig. 7C)

### Meta-analysis of umbilical artery pH, newborn PCO_2_ (kPa), PO_2_ (kPa), BE (mmol/L)

**Fig. 8:**
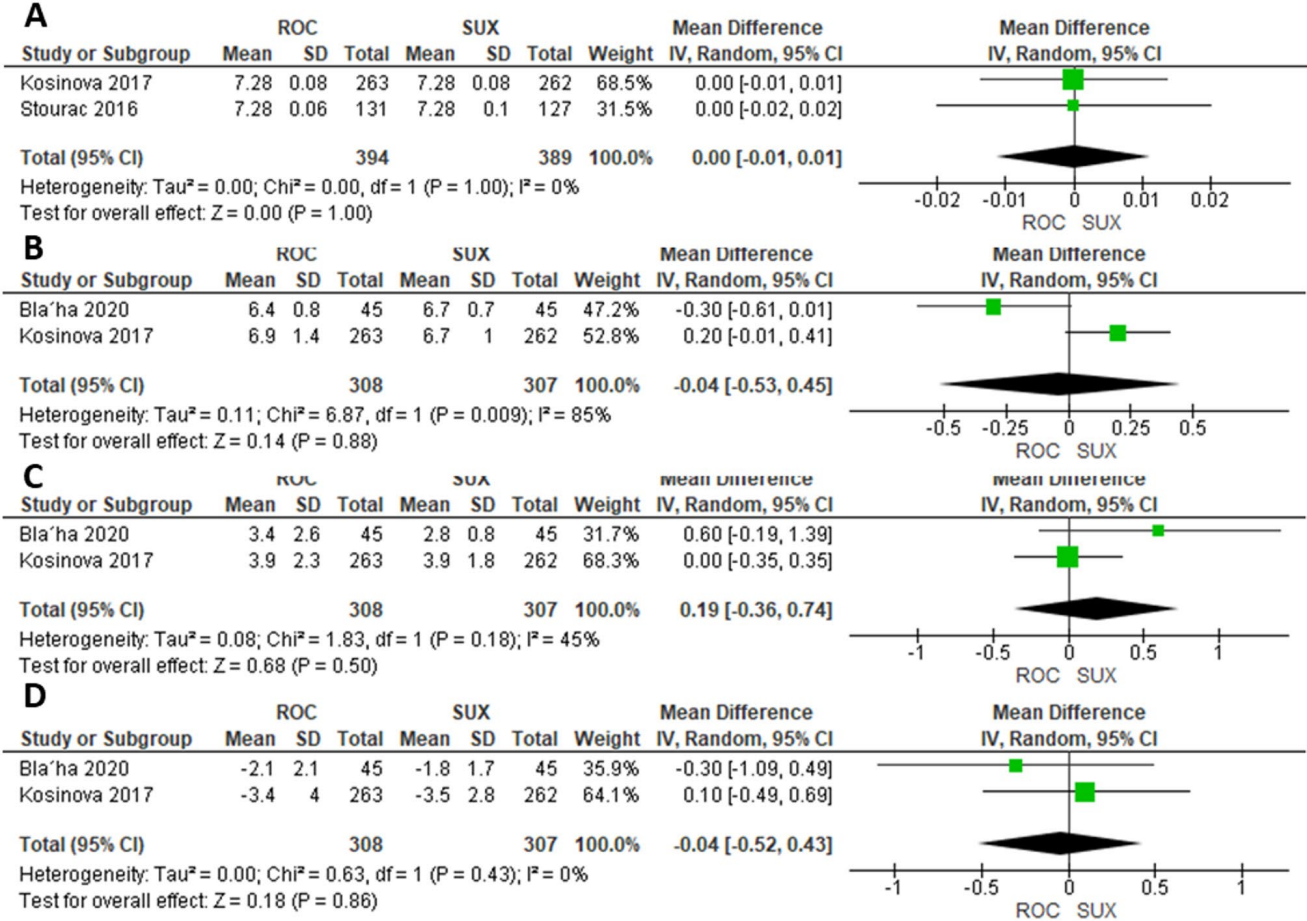
**A**) Umbilical Artery pH, **B**) Newborn PCO_2_, **C**) Newborn PO_2_, **D**) Newborn BE

Two studies were included in the meta-analysis comparing ROC (*n* = 394) and SUX (*n* = 389) for the mean umbilical artery pH that did not differ significantly between the ROC and SUX groups (MD = 0.00, 95% CI= [−0.01, 0.01]; *P* = 1.00; I²=0%) (Fig. 8A). Similarly, Two studies were included in the meta-analysis comparing ROC (*n* = 308) and SUX (*n* = 307) for Newborn PCO_2_ (kPa), PO_2_ (kPa), BE (mmol/L) that did not differ significantly between the ROC and SUX groups (MD= −0.04; 95% CI= [−0.53, 0.45]; *P* = 0.88; I²=85%, Fig. 8B), (MD = 0.19; 95% CI= [−0.36, 0.74]; *P* = 0.50; I²=45%, Fig. 8C), (MD= −0.04; 95% CI= [−0.52, 0.43]; *P* = 0.86; I²=0%, Fig. 8D), respectively.

### Publication bias

Assessing publication bias using the funnel plot and Egger’s test is unreliable for meta-analyses containing fewer than ten studies. Therefore, our data do not allow us to determine the presence or extent of publication bias.

## Discussion

The findings of this systematic review and meta-analysis provide valuable insights into the comparative efficacy and safety of ROC and SUX for general anesthesia in CS. The included studies, although limited in number, encompass a total of 1122 patients, offering a robust data set for analysis. However, the current evidence base has limitations, particularly since neonatal outcomes are often secondary data with no direct correlation to cord blood concentrations of ROC, as well as varying risk of bias, suggesting that the results should be interpreted with caution.

Substantial heterogeneity was observed across several outcomes, particularly for the time from incision to delivery (I² = 94%), surgery duration (I² = 63%), newborn weight (I² = 68%), and gestational age (I² = 95%). This heterogeneity may be attributed to methodological and clinical differences among the included studies. Stourac et al. (2016) included a large sample of 240 women aged 14–60 undergoing cesarean delivery, with both ROC and SUX administered at equipotent doses (1 mg/kg). However, the broad age range and potentially unstandardized obstetric or anesthetic protocols could introduce variability in delivery times and neonatal outcomes. In Bláha et al. (2020), the study population consisted of 90 women with anticipated difficult fetal delivery, and the anesthetic regimen included sugammadex 4 mg/kg for ROC reversal and neostigmine-atropine for SUX, which markedly differs from other studies. The lower dose of ROC (0.6 mg/kg) may also contribute to heterogeneity in intubation conditions and delivery time. Additionally, the inclusion of atracurium post-delivery in the SUX group introduces further confounding. Kosinova et al. (2017), the largest study (488 parturients), included 525 newborns, implying multiple births per mother, which could skew newborn weight and Apgar score data. Sheikh et al. (2008) conducted a smaller study (*n* = 50) that administered a higher SUX dose (1.5 mg/kg vs. 1 mg/kg in others) and employed thiopentone as the induction agent, which may have potentially affected intubation quality and neonatal acid-base balance. Furthermore, the exclusion of patients with anticipated difficult intubation and neuromuscular disease in this trial may have artificially improved outcomes in both groups, reducing generalizability. Collectively, variability in anesthetic regimens, muscle relaxant dosages, induction agents, reversal strategies, and patient characteristics (age, parity, predicted fetal weight, and comorbidities) are likely key contributors to the observed heterogeneity.

For many years, SUX has been the most commonly used agent for general anesthesia during caesarean Sections [14]. However, a 2021 survey revealed that the traditional use of SUX for rapid sequence induction (RSI) in obstetric anesthesia in the UK is gradually being supplemented by ROC. The survey data indicated a slight decrease in the dominance of SUX in obstetric general anesthesia compared to earlier surveys. In both the National Audit Project (NAP5) in 2013 and the 6th National Audit Project (NAP6) in 2016, SUX was used for tracheal intubation in over 90% of obstetric patients. In the 2021 study, it was used for 86% of patients, almost exclusively for RSI. The study also noted that drugs for reversing neuromuscular blockade were used in 88.1% of patients who received a non-depolarizing drug, an increase from the 68% reversal usage identified in NAP5 [15–17]. Current guidelines, published in 2015, suggest using ROC instead of SUX for neuromuscular blockade, although these guidelines are based on expert consensus rather than high-level evidence [18, 19].

Succinylcholine, a depolarizing neuromuscular blocking agent, is advantageous for rapid sequence intubation (RSI) because of its quick onset (40–60 s) and short duration of action (6–10 min). However, its benefits are limited by associated contraindications and adverse effects, such as hyperkalemia, malignant hyperthermia, and increased intraocular and intracranial pressure [20, 21]. The adverse effects of succinylcholine require extra caution in pregnant women because their levels of pseudocholinesterase can naturally decrease by up to 30% [22]. Another disadvantage that hypothetically might impact neonatal outcome is that SUX increases oxygen consumption through its depolarizing action, and hence may cause earlier desaturation compared to ROC [23].

In contrast, ROC has a longer onset (60–120 s) and a significantly longer duration of action (30–67 min) [24]. Another reason why ROC is becoming more widely used as a substitute for succinylcholine is the availability of a specific reversal agent Sugammadex [25]. Yet, Sugammadex cost and availability limits its use currently [18]. Another variable to consider about ROC dose is its placental transfer rate of 16% and its effect on the newborn well-being [26] while SUX does not cross the placenta when administered in usual clinical doses [19].

Multiple trials comparing the safety and efficacy of both agents have reported conflicting outcomes. Some studies reported ROC as non-inferior in achieving rapid sequence induction intubation [27], whereas another reported succinylcholine superiority [5]. This conflict is mostly related to the variation of ROC dose, use of opioids and hypnotics [5, 28]. Hwang et al. found that administering a high dose of ROC 1 mg/kg did not result in any maternal or fetal adverse effects compared to the conventional dose of 0.6 mg/kg [29]. However, other studies reported a statistically significant decrease in the 1-minute Apgar score with the high dose of ROC (1 mg/kg) [9]. Authors of another trial recommended even a higher dose of ROC 1.2 mg/kg reversed with Sugammadex 4 mg/kg. However, this was a small, uncontrolled investigation with limited neonatal outcome data [30].

Our results indicate that SUX may have advantages over ROC in certain aspects of anesthesia management during CS. Notably, the time from induction to clamping the umbilical cord was significantly shorter with SUX, which could be clinically important in minimizing fetal exposure to anesthetic agents. However, the time from incision to delivery and surgery duration show no significant differences between ROC and SUX.

Intubation conditions, a critical factor in the safety and efficacy of anesthesia, showed a mixed picture. Our analysis revealed that SUX was associated with better odds of achieving excellent intubation conditions, but ROC was linked to better odds of achieving good intubation conditions, respectively (Fig. 7A and B). Additionally, the odds of poor intubation conditions did not differ significantly between the ROC and SUX groups (Fig. 7C). This suggests that while SUX might be preferred for optimal intubation, ROC remains a viable alternative, especially when SUX is contraindicated. Succinylcholine is known advantageous for rapid RSI because of its quick onset (40–60 s) and short duration of action (6–10 min) compared to ROC longer onset (60–120 s) and a significantly longer duration of action (30–67 min) [24].

Neonatal outcomes also demonstrated some differences between the two agents. Our meta-analysis revealed that 1-minute and 5-minute Apgar scores favored SUX, the difference in the 10-minute Apgar score was not statistically significant. Even that Apgar score is an acceptable neonatal evaluation method, the relevance of a low 1-min Apgar score is uncertain [19]. These findings suggest that while SUX may confer some early benefits in neonatal well-being, these differences may not persist in the longer term. The current available trials provide neonatal outcomes as a secondary data with no correlation to cord blood concentrations of ROC with Apgar scores and neonatal neuromuscular function. The early disadvantage of ROC could be attributed to its 16% pacental transfer which could explain its effect on the newborn well-being [26] while SUX does not cross the placenta when administered in usual clinical doses [19].

The Apgar score remains a useful immediate assessment of neonatal status but has important limitations. A low 1-minute score often reflects the transient effects of maternal anesthesia, residual neuromuscular blockade, or adaptation to extrauterine life, and is only weakly predictive of long-term outcomes [19, 31]. In contrast, the 5-minute Apgar score correlates more closely with risks of neonatal mortality, hypoxic–ischemic encephalopathy, and cerebral palsy when it is below 7, and thus carries greater prognostic value [31, 32]. By 10 min, an improving Apgar score usually indicates recovery, whereas persistently low scores (≤ 3) are associated with significant neurologic impairment and higher mortality [31, 32]. In our analysis, the advantage of succinylcholine at 1 and 5 min suggests better early cardiorespiratory adaptation, but the lack of difference at 10 min implies that both agents ultimately allow similar neonatal recovery. Given these nuances, Apgar scores should be interpreted in conjunction with other measures, such as cord blood gas analysis or longer-term neurodevelopmental follow-up, to assess neonatal safety in cesarean anesthesia studies fully.

Future research should explore the relationship between cord blood concentrations of ROC and neonatal outcomes, such as Apgar scores and neuromuscular function. This is particularly relevant given that a 2021 survey found SUX was used in 86% of cases for rapid sequence induction in obstetric anesthesia, compared to over 90% in national surveys conducted in 2013 and 2016 in the UK [15–17].

The meta-analysis also examined other neonatal parameters, including umbilical artery pH, PCO_2_, PO_2_, and base excess, with no significant differences observed between the two groups. This reinforces the idea that both agents are comparable in terms of their impact on neonatal blood gas parameters, which are crucial indicators of fetal well-being.

This meta‑analysis has some limitations. First, only six studies were available for inclusion, yielding a modest overall sample size and precluding robust subgroup analyses. Second, significant heterogeneity was observed for several outcomes, such as incision-to-delivery time, newborn weight, and gestational age, likely reflecting differences in neuromuscular blocker dosing, induction agents, reversal strategies, and patient characteristics across trials. Third, key safety endpoints such as maternal adverse events (failed intubation, aspiration, hemodynamic instability) and detailed neonatal clinical outcomes beyond Apgar scores were not consistently reported and therefore could not be pooled. Finally, most neonatal outcomes were reported as secondary endpoints without concurrent cord‑blood drug concentrations or long‑term follow‑up, limiting our ability to draw definitive conclusions about fetal exposure and neurodevelopmental impact. These limitations underscore the need for larger, well‑powered RCTs with standardized protocols and comprehensive safety reporting to strengthen evidence for obstetric anesthesia practice.

## Conclusion

This systematic review and meta-analysis revealed that while SUX shows some advantages, such as shorter induction-to-cord clamping times and better early Apgar scores, ROC remains a viable alternative, especially in situations where SUX may be contraindicated. Neonatal outcomes also demonstrated some differences between the two agents. The meta-analysis revealed that 1-minute and 5-minute Apgar scores favored SUX, the difference in the 10-minute Apgar score was not statistically significant. However, the current evidence base has limitations, particularly since neonatal outcomes are often secondary data with no direct correlation to cord blood concentrations of ROC, Apgar scores, or neonatal neuromuscular function. Therefore, additional well-designed RCTs are necessary to draw firmer conclusions. These findings highlight the importance of future research that considers the impact on both mother and child, emphasizing the need to optimize outcomes for both.

## Supplementary Information

Below is the link to the electronic supplementary material.

Supplementary Material 1

## Data Availability

All data generated or analysed during this study are included in this published article [and its supplementary information files].

## Abbreviations

ROC: Rocuronium
SUX: Suxamethonium
ASA PS: American Society of Anesthesiologists Physical Status
BMI: Body Mass Index
N/A: Not Available

## Declarations

### Generative AI and AI-assisted technologies in the writing process

During the preparation of this work the author(s) used ChatGPT in order to improve language. After using this tool/service, the author(s) reviewed and edited the content as needed and take(s) full responsibility for the content of the publication.
