# Supplementary material for: Intubation conditions and neonatal outcomes with rocuronium versus suxamethonium in cesarean sections: A systematic review and meta-analysis
Source: BMC Anesthesiol. 2025 Oct 2;25:476. doi: 10.1186/s12871-025-03321-6 (PMC12490032; doi:10.1186/s12871-025-03321-6)
Supplement: Supplementary file 1 — Supplementary Material 1 [file 12871_2025_3321_MOESM1_ESM.docx]

# **Table 1 Search strategies**

| Database | Query | N |
| --- | --- | --- |
| PubMed | ("Rocuronium"[Mesh] OR rocuronium OR "Rocuronium Bromide") AND  ("Succinylcholine"[Mesh] OR suxamethonium OR "Suxamethonium Chloride") AND  ("Cesarean Section"[Mesh] OR "Cesarean Delivery" OR "C-section" OR "Caesarean") | 21 |
| Cochrane CENTRAL | (rocuronium OR "rocuronium bromide") AND  (suxamethonium OR succinylcholine) | 344 |
| Web of Science | (rocuronium OR "rocuronium bromide") AND  (suxamethonium OR succinylcholine) AND  ("cesarean section" OR "cesarean delivery" OR caesarean) | 45 |
| Scopus | TITLE-ABS-KEY(rocuronium OR "rocuronium bromide") AND  TITLE-ABS-KEY(suxamethonium OR succinylcholine) AND  TITLE-ABS-KEY("cesarean section" OR "cesarean delivery" OR caesarean) | 174 |
